# Depositional Environment and Ecological Response of Bioconstructions: A Case Study of Southern China (Guizhou Province) in Moscovian–Gzhelian

**DOI:** 10.3390/life14091150

**Published:** 2024-09-11

**Authors:** Xiao Li, Enpu Gong, Yongli Zhang, Changqing Guan, Wentao Huang

**Affiliations:** 1 College of Resources and Civil Engineering, Northeastern University, Wenhua Road 3-11, Heping District, Shenyang 110819, China; 1710351@stu.neu.edu.cn (X.L.); guanchangqing@mail.neu.edu.cn (C.G.); 2 Development Research Center, China Geological Survey, Fuwai Street 45, Xicheng District, Beijing 100037, China; wentao1990@126.com

**Keywords:** skeletal grains, biostrome, quantitative analysis, canonical correspondence analysis, theoretical ecospace, depositional settings, microbial bioconstruction, Pennsylvanian, south China

## Abstract

From the late Carboniferous to the early Permian, multiple pulses of glaciation and deglaciation have been caused by the LPIA. The Pennsylvanian period experienced phases of recovery, proliferation, and decline, ultimately forming a reef system distinctly different from that of the Mississippian period. During the late Bashkirian to Moscovian, the metazoan reef experienced a limited resurgence, with reef predominantly formed by chaetetid developing in the United States, northern China, and Japan. During the Kasimovian to Gzhelian, the phylloid algal reef dominated the global reef systems. In the late Pennsylvanian, bioconstruction cases and paleoenvironmental proxies in southern Guizhou Province were studied to investigate the composition, recovery, and evolutionary processes of the bioconstructions as well as their response to environmental variations during this period. Several bioconstructions have been reported in the Lumazhai section of Houchang Town, Guizhou Province, southern China, from the Moscovian to the Gzhelian. The upper Carboniferous strata are well-preserved and continuously exposed. The continuous strata, abundant fossils, and diverse bioconstructions provide excellent research materials for exploring the mutual constraints between organisms and their environment. This study identified ten microfacies, whose vertical evolution indicated significant changes in the depositional environment related to relative sea-level fluctuations. Skeletal grains are widely present in these facies. Among them, foraminifera, algae, bryozoans, crinoids, and *Tubiphytes* are the most common and exhibit distinct distribution characteristics in various environments. Quantitative statistics, CCA and theoretical ecospace have been utilized to examine and interpret environmental impact factors. Quantitative analysis of their relative abundance and distribution patterns provides insights into the complex interactions between organisms and environmental factors. The relative abundances of different organisms and factors controlling their bioconstructions are influenced by relative sea-level changes. CCA analysis reveal that hydrodynamic conditions are the primary influencing factor. Variation trends in average tiering and motility reveal the characteristics of biological communities during environmental changes in phylloid algae and microbial bioconstructions. These bioconstructions are not directly correlated with changes in environmental factors, and the biological communities in phylloid algae mounds and biostromes exhibit similar organism compositions and ecological niches across different environments.

## 1. Introduction

The Late Paleozoic Ice Age (LPIA; ~360–260 Ma) was a pivotal period in geological history, marking significant global climate changes that profoundly influenced the paleoclimate, paleoenvironments, and paleoecology [1,2,3,4,5]. Due to paleogeographic constraints, temperatures in low-latitude regions rarely decreased below freezing year-round, preventing the formation of glacial deposits. However, glacial episodes coincided with fluctuations in sea levels and biosphere dynamics, impacting sensitive reef ecosystems known for their intricate ecological systems [6,7,8,9]. The number and scale of Carboniferous reefs, impacted by extinction events of the Late Devonian, were significantly smaller compared to those of the Devonian and Permian. Following a sharp decline in Carboniferous reefs by the late Mississippian, there was a localized resurgence of metazoan reefs from the late Bashkirian to the Moscovian [10,11]. 

The Carboniferous period was characterized by significant reef system evolution and exhibited notable parallels with the Late Paleozoic Ice Age [9]. The Pennsylvanian represents a major phase in the Late Paleozoic Ice Age, with the widespread cyclic deposits in marine strata indicating frequent fluctuations in sea level [2,5,9]. Toward the end of the Moscovian, a minor global biotic extinction event marked the disappearance of coral and chaetetid reef communities, which were subsequently dominated by phylloid algae until the late Moscovian to Gzhelian [10]. Previous studies on reefs during different ice ages have focused primarily on factors such as reef quantity, scale, and key reef-building organisms. However, detailed investigations into the distribution patterns and ecological spatial changes of various organisms during their development processes remain limited. Studies have suggested that marine ecosystems often undergo gradual reconstruction from lower to upper trophic levels, representing a stepwise recovery process. Exploring triggers for biological recovery is crucial to elucidate the intricate interactions and balances between organisms and their environment.

This study focused on the Lumazhai section near Houchang Town, Ziyun, Guizhou, which is renowned for its diverse bioconstructions. In addition to coral reefs, the Lumazhai section features coral biostrome, microbial mounds, and multiple phylloid algae biostromes. The bioconstructions of the Lumazhai section provide an ideal site to study the initiating conditions for reef community. The continuous strata, abundant fossils, and diverse bioconstructions provide excellent research materials for quantitative statistics. The aim of this work was to comprehensively investigate the biological characteristics of basal ecological functional groups during the late Carboniferous, reconstruct the biological landscape of basal ecosystems, and offer empirical evidence to explore the mutual constraints and balances between organisms and their environment.

## 2. Geological Settings

The upper Carboniferous to Permian strata in South China are well-preserved and continuously exposed, characterized by an extended shallow carbonate platform comprising the Yangzi, Southwest, South Central, Longmen Shan, and Western Yunnan platforms [12]. The Pennsylvanian was a period of significant global paleogeographic changes. The Rheic Ocean, located between the Laurussian and Gondwanan continents, began to close during the late Mississippian period. Starting from the Pennsylvanian, the gradual collision of Laurussia and Gondwana led to the formation of Pangea, with the Paleo-Tethys Ocean to its east and the Panthalassa Ocean to its west [13].

Strata from the Upper Carboniferous to the Permian are well-preserved in the study area, which are representative of shallow marine deposits composed of light grey, medium- to thick-bedded massive limestones [12]. The Upper Carboniferous sequences, consisting of Weining and Maping formations, contained abundant fossils [12]. Reefs developed extensively during the late Carboniferous in Guizhou. Numerous bioconstructions have been previously studied including microbial mounds, sponge reefs, phylloid algae reefs, *Tubiphytes* reefs, and coral reefs [14,15,16,17,18]. 

**Figure 1:**
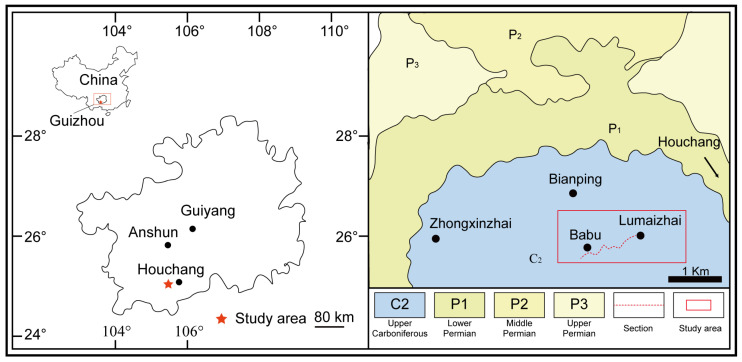
A geological map of the study area and location of the Lumazhai section.

**Figure 2:**
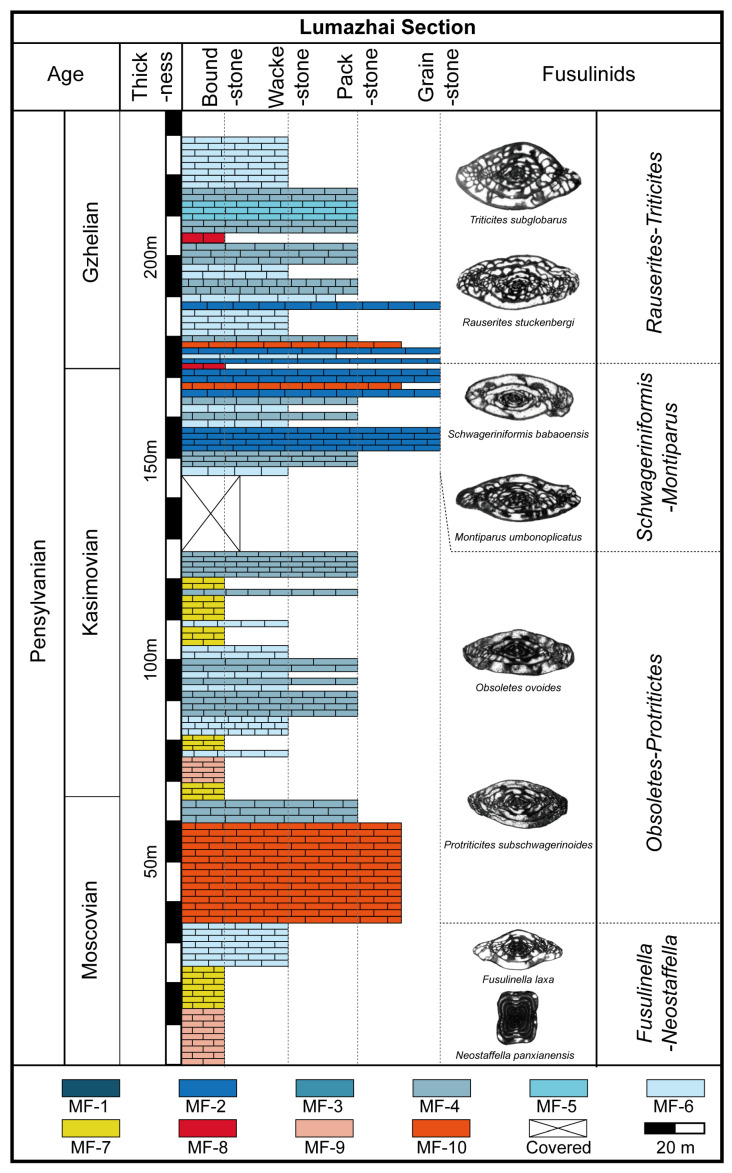
Stratigraphic column distributions of the microfacies types in the Lumazhai section and fusulinid zone.

The Lumazhai section is situated within the Southwest platform and is positioned at the margin of the Luodian intraplatform basin. It is a continuous outcrop, located near Lumazhai Village, in Houchang Town, Ziyun County, southern Guizhou Province (Figure 1). This section has a thickness of approximately 272 m, composed mainly of thick-bedded grey limestone, and contains a rich assemblage of benthic organisms along with several bioconstructions (Figure 2). The section can be subdivided into eight lithologic beds. Bed 1 (from 0 to 34 m) is dominated by microbial boundstone and phylloid algae boundstone. Bed 2 (from 34 to 77 m) is composed of bioclastic wackestone, packstone, and grainstone. Bed 3 (from 77 to 166 m) is composed of five phylloid algae biostromes and one microbial biostrome alternating with bioclastic packstone. Bed 4 (from 166 to 191 m) is characterized by bioclastic packstone and grainstone. Bed 5 (from 191 to 191.8 m) is coral biostrome. Bed 6 (from 191.8 to 233 m) is dominated by bioclastic wackestone and packstone. Bed 7 (from 233 to 233.5 m) is the second coral biostrome. Bed 8 (from 233.5 to 272 m) is composed of bioclastic wackestone and packstone. Fusulinids are widely distributed throughout the section, and a comprehensive study of fusulinids was carried out (Figure 2). *Fusulinella helenae*, *F*. *helenae*, *F*. *praebocki*, *Neostaffella cuboides*, *N*. *greenlandica*, and *N*. *panxianensis* were recognized in the *Fusulinella*-*Fusulina* Zone, establishing the age of beds 1–3 as late Moscovian [19]. The occurrence of *Protriticites ziyunensis*, *P*. *subschwagerinoides*, *P*. *globulus*, *Montiparus longissima*, *M*. *huishuiensis*, *M*. *reticulatus*, *Schwagerinformis minor*, *S*. *nanus* in beds 4–7 indicate an early Kasimovian age [19,20]. The widespread presence of *Triticites longissima*, *T*. *nadezhdae*, *Rauserites shikhanensis variabilis* in beds 8–15 represents a Gzhelian age [19,20]. In summary, fusulinids in this study suggest a late Moscovian-Gzhelian age.

## 3. Methods and Database

**Figure 3:**
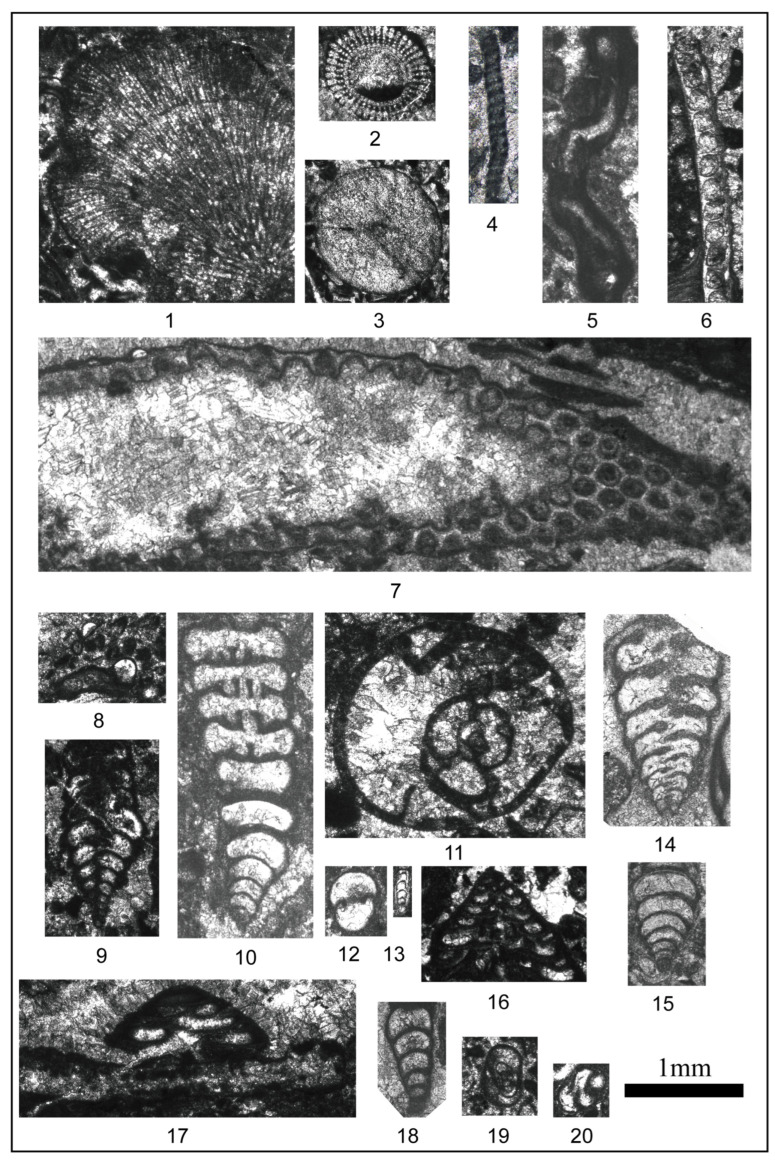
Photographs of organisms in the section. **1** *Parachaetetes*, **2**–**3** Echinoderm, **4** *Beresella*, **5** *Tubiphytes*, **6** Bryozoan, **7** Epimastoporelleae, **8** *Tubertina*, **9** *Palaeotextularia*, **10** *Cribrogenerina*, **11** *Bradyina*, **12** *Neotuberitina*, **13** *Protonodosaria*, **14** *Palaeotextularia*, **15** *Climacammina*, **16**–**17** *Tetrataxis*, **18** *Climacammina*, **19** *Bradyina*, **20** *Globivalvulina*.

To obtain quantitative data, the data primarily involves two aspects on the basis of the different types of fossils. First, for countable large fossils such as corals and brachiopods, statistical analysis is conducted during field section surveys. Second, for small fossils such as foraminifera, bryozoans, crinoids, phylloid algae, and microbes that cannot be directly quantified, microscopic analysis is used (Figure 3).

**Figure 4:**
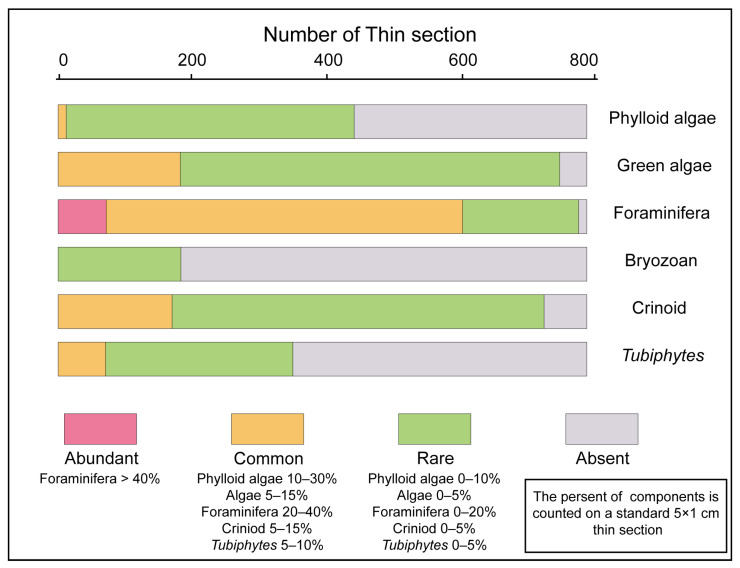
The relative abundance in thin sections examined.

Over 800 thin sections were examined via a petrographic microscope. For each thin section, 300 points were counted with a petrological microscope to determine the proportions of matrix, cement, nonskeletal clasts, and skeletal components. However, detailed analysis of the genus and species diversity is of limited utility for statistical comparison due to the disproportionate number of variables and their scant representation in the database. Furthermore, because of the presence of some large organisms such as brachiopods and corals as well as the difficulty in quantifying certain microorganisms, it becomes challenging to compare their abundances with those of organisms on the same scale. Consequently, the most diverse skeletal components were categorized into distinct groups including foraminifera, phylloid algae, green algae, bryozoans, crinoids, calcispheres, and *Tubiphytes* (Figure 4).

Since each stratigraphic level is represented by several thin sections, typically ranging from four to eight, the percentages were recalculated to 100% for a better comparison. Internal fillings or cements within moderate to large shells were quantified as matrix or cement, as observed in large corals.

Multivariate statistical analysis is a methodology used to investigate the interrelationships and interactions among multiple variables. It is widely applied in ecology, biology, and geology [21,22,23,24]. This method helps researchers extract crucial information from extensive datasets, discover potential patterns and trends, identify significant variables, and support hypothesis validation and theory development. Canonical correspondence analysis (CCA) is one of the most extensively used techniques in ecological studies. It evaluates quantitative variables with respect to the qualitative variables within each main cluster, enabling the comparison of quantitative variables against well-established environmental parameters. To reduce noise in the database, samples present in less than 5% of the database are not given significant influence during the analysis [25]. Consequently, only a portion of the database is used in the assessment. Identifying the controlling factors governing the organism–environment relationship in the CCA is largely based on paleoecological dependencies and interpretations.

**Figure 5:**
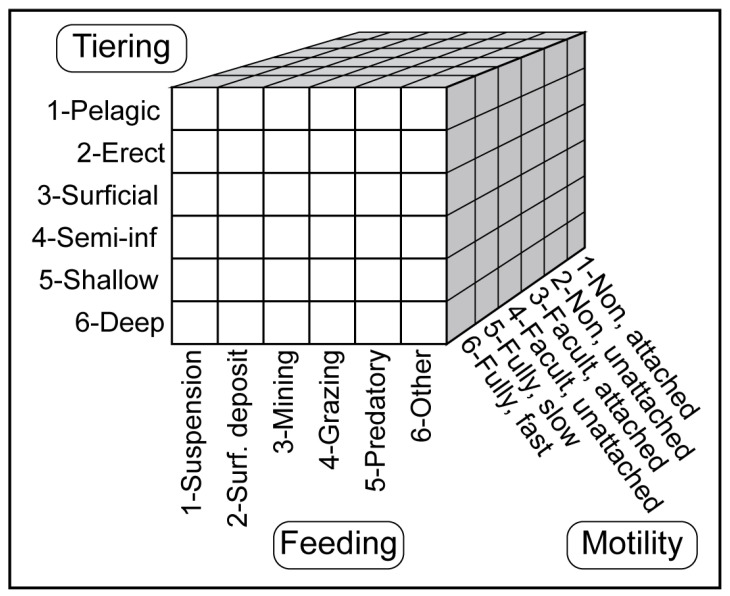
Ecospace as defined by the three axes of tiering, motility level, and feeding strategy [26].

Bambach [26] proposed a method for marine ecological classification using theoretical ecospace on the basis of three aspects: tiering, motility, and feeding strategy (Figure 5). Each aspect is subdivided into six categories, resulting in 216 potential life modes. Tiering is categorized as pelagic, erect, surficial, semi, shallow, or deep. Motility is divided into fast fully motile, slow fully motile, unattached facult motile, attached facult motile, unattached non-motile, and attached non-motile. In the theoretical ecospace, the ecological types for tiering and motility are coded from 1 to 6 in an ordered manner. Tiering codes from 1 to 6 indicate a gradient from higher to lower tiering, with living positions changing from above the substrate to below it. A lower tiering code indicates a higher living position. Motility codes from 1 to 6 represent a gradient from rapid movement to immobility.

## 4. Results

### 4.1. Lithofacies Types

**Figure 6:**
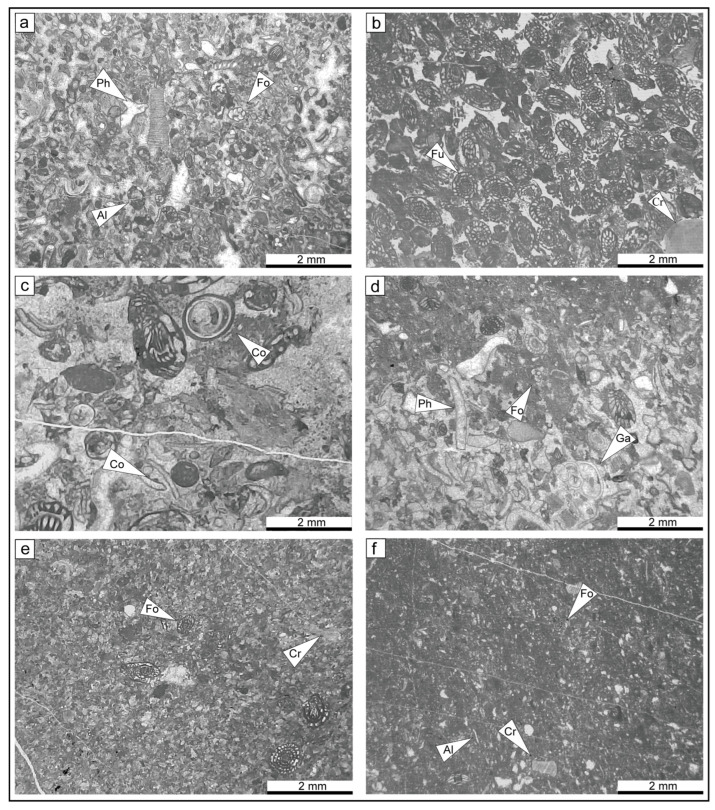
Photographs of the MF-1 to MF-6 lithofacies types. (**a**) MF-1, (**b**) MF-2, (**c**) MF-3, (**d**) MF-4, (**e**) MF-5, (**f**) MF-6. Ph—phylloid algae; Fo—foraminifera; Al—algae; Fu—fusulinids; Cr—crinoid.

**Figure 7:**
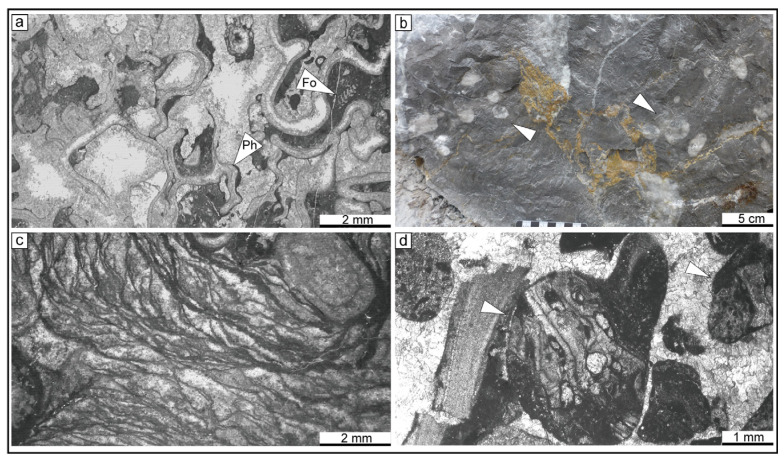
Photographs of the MF-7 to MF-10 lithofacies types. (**a**) MF-7; (**b**) MF-8, *Fomichevella* coral (white arrow); (**c**) MF-9; (**d**) MF-10, interpretative sketch (white arrow).

The measured section consists of a series of shallow-water carbonates. Several types of bioconstructions are found in the section including microbial mounds, microbial-phylloid algae biostrome, phylloid algae biostromes, and coral biostromes. The microfacies are described below (Figure 6 and Figure 7).

MF-1 Bioclastic grainstone

**Description** The facies consists of abundant, well-sorted, and well-rounded grainstone. The skeletal grains primarily consist of fusulinids, foraminifera, and algae. Among them, foraminifera account for 30% to 40%, green algae account for approximately 10%, micrite accounts for less than 15%, and cement accounts for more than 30%.

**Interpretation** The skeletal association, with a low content of micrite, well-rounded and sorted grains, and predominant texture, suggests a high-energy environment on a shallow shoal at or above the fair-weather wave base. It is equivalent to SMF-18 and FZ 7–8 of Flügel [27].

MF-2 Foraminiferal grainstone

**Description** The facies is characterized by abundant foraminifera that are commonly associated with green algae, crinoids, *Tubiphytes*, gastropods, bryozoans, and coral debris. The skeletal grains partially exhibit micrite envelopes. Mud clasts and grain aggregates are common. 

**Interpretation** Diverse skeletal organisms suggest an open-marine depositional environment. The association of green algae and *Tubiphytes* indicates a marine setting within the euphotic zone [27,28,29]. The presence of cortoids and relatively high cement indicates medium to high hydrodynamic conditions, at or below the fair-weather wave base.

MF-3 Coated-grain grainstone

**Description** Coated-grain grainstone is characterized by abundant cortoids (30–50%) ranging in size from 0.5 to 3 mm. The biogenic components consist of foraminifera, crinoids, bryozoans, brachiopods, *Tubiphytes*, green algae, and phylloid algae fragments along with scarce mud clasts (0.5–2 mm in diameter). Block, drusy, and radixial-fibrous calcite cements are common. The micritic matrix is rare (<10%). 

**Interpretation** Cortoids result from the destructive and constructive processes linked to the activity of microboring organisms, which is light-dependent [27]. These coated grains are often interpreted as indicators of a shallow marine environment [30,31,32]. Additionally, green algae point to an environment within the photic zone. The microfacies are equivalent to SMF 11 and FZ 5–6 corresponding to shallow water, with normal marine salinity, and constant wave action, which are located at or above the FWWB in the photic zone [27]. 

MF-4 Bioclastic packstone

**Description** Bioclastic packstone-grainstone is widely distributed in the Lumazhai section and is characterized by an abundance of angular fragments of skeletal components. Various bioclasts including foraminifera, crinoids, algae, corals, calcimicrobes, brachiopods, *Tubiphytes*, phylloid algae, and bryozoans make up about 40–70% of the bioclasts, ranging in size from 0.1 to 0.5 cm. Peloids, mud clasts, and coated grains can be observed in the microfacies. 

**Interpretation** Phylloid algae are commonly reported as growing in shallow waters within the photic zone [33,34,35]. Dasyclad green algae usually grow in shallow water [28]. The occurrence of suspension feeders such as bryozoans and corals suggests well-oxygenated waters, normal salinity, and open-marine circulation [36]. 

MF-5 Bioclastic packstone with abundant crinoids

**Description** This facies is composed of more than 40% abundant crinoids. Other bioclasts include rare foraminifera. The crinoids consist almost exclusively of isolated, often worn disks and plates. The facies is poorly washed and contains more than 60% micritic matrix. 

**Interpretation** Broken crinoid fragments and the absence of other organisms may indicate in-place degradation or deposition subsequent to transport [27]. Poor sorting and rounding of grains indicate a limited transportation. The random orientation of the grains and poor sorting with the poor preservation of crinoids is considered the sedimentation below the fair-weather wave base and within the storm wave zone [37]. Thus, this facies indicates the sedimentation on the slope, below the fair-weather wave base, and above the storm wave base with low water energy [27].

MF-6 Bioclastic wackestone

**Description** The major elements of this microfacies are bioclasts (10–20%) including foraminifera, *Tubiphytes*, crinoids, bryozoans, and brachiopod fragments. It is cemented by sparite (10–20%) with micritic mud (50–80%). The bioclasts are well-preserved. Lithoclasts and coated grains are rare in this facies.

**Interpretation** Green algae suggest a photic depositional environment [28]. The abundance of micritic mud and the absence of green algae indicate low to moderate hydrodynamic conditions. The association of bryozoans with brachiopods attests to well-oxygenated waters and normal salinity [36]. Thus, the bioclastic wackestone-packstone originated in an open lagoon or open marine environment between the fair-weather wave base and storm wave base, in a dysphotic zone with low energy. 

MF-7 Phylloid algae boundstone

**Description** Phylloid algae form a dense framework and are commonly recrystallized. The leaf-like algae thalli are well-preserved with shelter cavities that are filled with marine cement. Other organisms such as foraminifera, echinoderms, green algae, bryozoans, *Tubiphytes*, and brachiopods are not abundant.

**Interpretation** Phylloid algae are usually reported in shallow water environments, within the photic zone, and under moderate energy conditions [34,38]. The various bioclasts and the association of green algae and *Tubiphytes* suggest an open marine environment with well-oxygenated waters [28,36]. It is equivalent to SMF 5 [27].

MF-8 Coral boundstone

**Description** Coral boundstone is constructed by *Fomichevella*, which represents approximately 20% of the rock volume, and reaches a maximum diameter of 4 cm, forming a distinct framework. Most of the corals are well-preserved and primarily rest on their sides or grow at a small angle. Primary cavities with sparry calcite are common. The matrix among corals consists of micrite and fragments of crinoids, green algae, and bryozoans. 

**Interpretation** Enclosing sediments composed of relatively high carbonate mud contents with scarce skeletal grains suggest low-energy conditions within deeper water [39,40]. Therefore, the coral boundstone developed in a low-energy depositional environment below the FWWB.

MF-9 Microbial boundstone

**Description** Microbial boundstone is characterized by stromatolitic textures, microbial fabrics, encrustation, and wrinkle structures on the weathered surface of the rock. Abundant marine cement occurs in the microbial boundstone. The calcimicrobes are mainly composed of *Gervanella*, *Ortonella*, and *Wetheredella*-like. Additional organisms include phylloid algae, foraminifera, corals, crinoids, bryozoans, and algae. 

**Interpretation** The association of microbial boundstone with algae and coral may indicate that it has been deposited in the euphotic zone. The occurrence of bryozoans and corals indicates well-oxygenated water with normal salinity [36]. All of these elements constrain the depositional environment from a platform margin to an upper slope environment with active water circulation within the euphotic zone.

MF-10 Lithoclastic rudstone

**Description** The lithoclastic rudstone is characterized by lithoclasts ranging from 0.5 to 2 mm in size. The main content of the clasts includes phylloid algae boundstone and bioclastic packstone debris. The grains are poorly sorted and subrounded. 

**Interpretation** The debris and the outlines of the clasts may imply transport and redeposition. The lack of micrite and the existence of depositional marine cement suggest relatively high energy. Lithoclastic rudstone is commonly found in slope settings [41,42]. 

**Figure 8:**
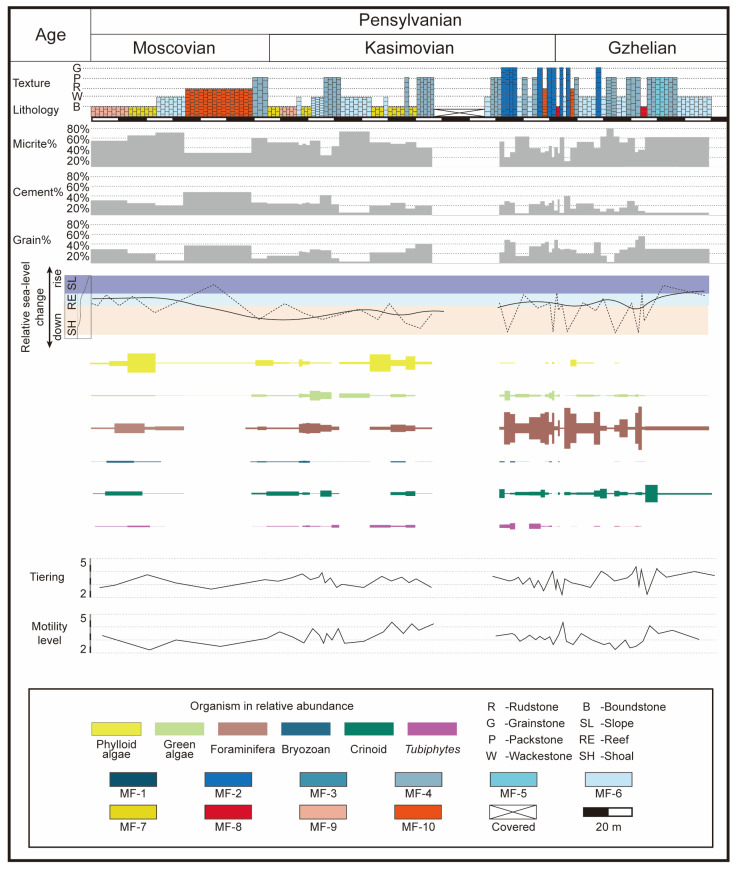
The sedimentary evolution, organism distribution, average tiering, and average motility level data of Lumazhai section.

Generally, the Lumazhai section is characterized by bioclastic limestone. The absence of siliciclastic deposits indicates a marginal shallow platform setting with low sediment influx. The presence of microbial boundstone is commonly interpreted as occurring from the platform margin to the upper slopes [27,28,43,44,45]. Additionally, the well-sorted foraminiferal grainstone may have accumulated on a margin shoal under turbulent water conditions. On the basis of the description and interpretation of the facies, the Lumazhai section suggests a shallow marine environment within the euphotic zone, likely on the platform margin (Figure 8).

### 4.2. Main Skeletal Grains and Distribution in Facies

Statistical data revealed significant variations in the abundances of foraminifera, phylloid algae, green algae, crinoids, bryozoans, and *Tubiphytes* that were observed in the Lumazhai section. A comparison of their distributions in different microfacies revealed distinct spatial patterns for these skeletal grains (Figure 8). 

Phylloid algae, prevalent from late Moscovian to late Kasimovian, play a crucial role in the formation of bioconstructions [34,46]. Globally, they hold a dominant ecological position in reef ecosystems from the Moscovian to Gzhelian [34,38,46,47]. In the Lumazhai section, phylloid algae are important because they contribute to the development of phylloid algae mounds and multiple phylloid algae biostromes. As skeletal grains, they exhibit distinct distribution patterns. Statistical data revealed that phylloid algae range from rare to common in the packstone and wackestone facies, whereas they were relatively rare in the grainstone facies. During the Moscovian to Kasimovian, when packstone and wackestone facies dominated, phylloid algae were abundant and widely distributed. However, during the Gzhelian period, which was characterized by frequent environmental changes and the prevalence of grainstone facies, their occurrence frequency decreased.

Green algae are commonly used as environmental indicators to infer conditions within the euphotic zone [28]. They exhibit a wide distribution across all facies, with occurrences ranging from rare to common in more than 94% of the thin sections. Additionally, within the phylloid algae biostromes, the abundance of green algae is relatively high. Interestingly, the abundance of wackestone, packstone, and grainstone microfacies can vary from high to low without a clear pattern. *Tubiphytes* exhibit varying abundance, ranging from rare to common in packstone to grainstone microfacies. Bryozoan fragments are relatively rare, appearing in fewer than 23% of the thin sections. In the Lumazhai section, bryozoans are predominantly found in boundstone and wackestone facies, whereas their occurrence in packstone and grainstone facies is rare. In contrast, crinoids have a wide range of occurrence, varying from rare to common in wackestone to grainstone microfacies, and represent the dominant bioclast in the MF-5 microfacies.

Foraminifera are important proxies used in paleoenvironmental and paleogeographic reconstructions [48]. They are widely distributed throughout the Lumazhai section, exhibiting relatively high abundance and notable variation. Therefore, a classification discussion of their classification is necessary. 

These foraminifera display a unique variety of test shapes and coiling modes linked to feeding strategies and environmental influences [49]. Their repeated occurrence in different wall structure groups indicates adaptive convergence [48,50,51,52,53,54,55]. Thus, a specific coiling mode can accommodate multiple feeding strategies, resulting in a complex relationship between morphology and behavior [49].

The concept of morphogroups, introduced by Jones and Charnock [56], aids in interpreting the significant factors controlling the eco-sedimentary environment [57,58,59]. Studies of foraminiferal assemblages suggest that test characteristics are directly related to their lifestyles and trophic strategies [59,60,61]. Consequently, some studies have utilized analogous morphological groupings in fossil assemblages to infer paleoenvironmental conditions [57,58,61,62,63,64].

**Figure 9:**
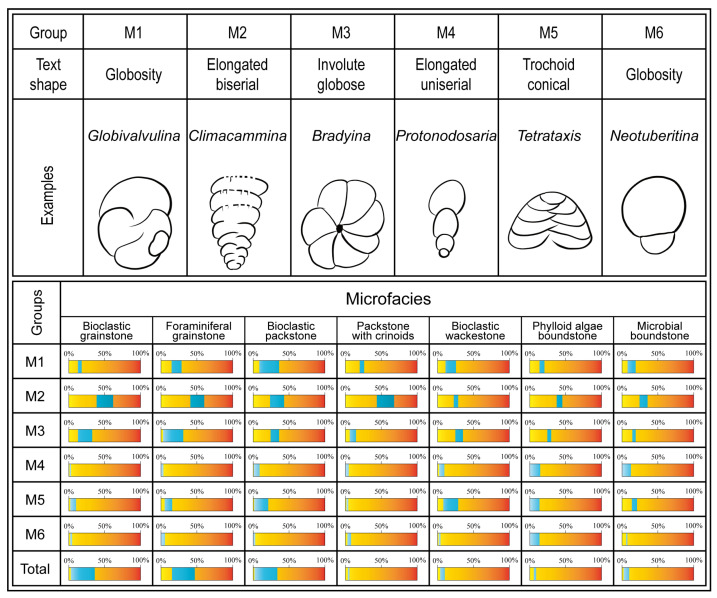
The classification of morphogroup and distribution in different microfacies. The blue portion represents the range of relative abundance within microfacies.

Following the relevant proposal for foraminifera, microfacies, and fossil assemblages, six morphogroups (Figure 9) were differentiated according to morphological features [51,55,56,57,61,65,66]. The M1 group comprises foraminifera with a globosity test and thick, multilayered walls. The representative genus is *Globivalvulina*. This group is interpreted as having an epifaunal to shallow infaunal habit [62]. The M2 group consists of elongated and biserial forms. The representative genera are *Palaeotextularia* and *Climmacamina*. It is comparable to morphogroups C1 and C3, as proposed by Jones and Charnock [56] and Reolid [55], with shallow to deep infaunal lifestyles and herbivorous and bacterial scavenger feeding habits. The foraminifera in the M3 group include involute globose and planispiral forms. Some genera in this group may live in shallow environments because their thick walls, globose tests, and sieve-like apertures could have protected them from damage in high-energy environments [50]. The representative genus is *Bradyina*. The test structure is similar to that of morphogroups B3, B4, and D proposed by Murray [54]. The M4 group is composed of elongated and uniserial foraminifera with thin walls and simple textures. The representative genus is *Protonodosaria*. The M5 group comprises trochoid conical tests with a shallow infaunal, epifaunal, and attached life habitats [67]. The representative genus is *Textraxis*. They are interpreted as deposit-feeders and detritivores [48,55,61]. The characteristic test shape easily attached to substrates and promoted stability in turbulent environments [67]. The M6 group is composed of globosity foraminifera with two small spherical chambers. The representative genus is *Neotuberitina*, which was adapted to epifaunal habitats with deposit feeder and detritivore feeding strategies [51,59]. Spherical foraminifera occurred in both shallow and hypoxic water [51] and are considered opportunistic taxa [68].

With the use of biodiversity and abundance data, along with the microfacies analysis, the quantitative content of each morphogroup within distinct microfacies was determined (Figure 9). The proportions of M1, M4, and M6 consistently fell within similar intervals across all microfacies, showing minimal disparity between the maximum and minimum values. In contrast, the M2, M3, and M5 groups exhibited distinctive patterns of relative abundance and variability among the different microfacies.

### 4.3. Canonical Correspondence Analysis

**Figure 10:**
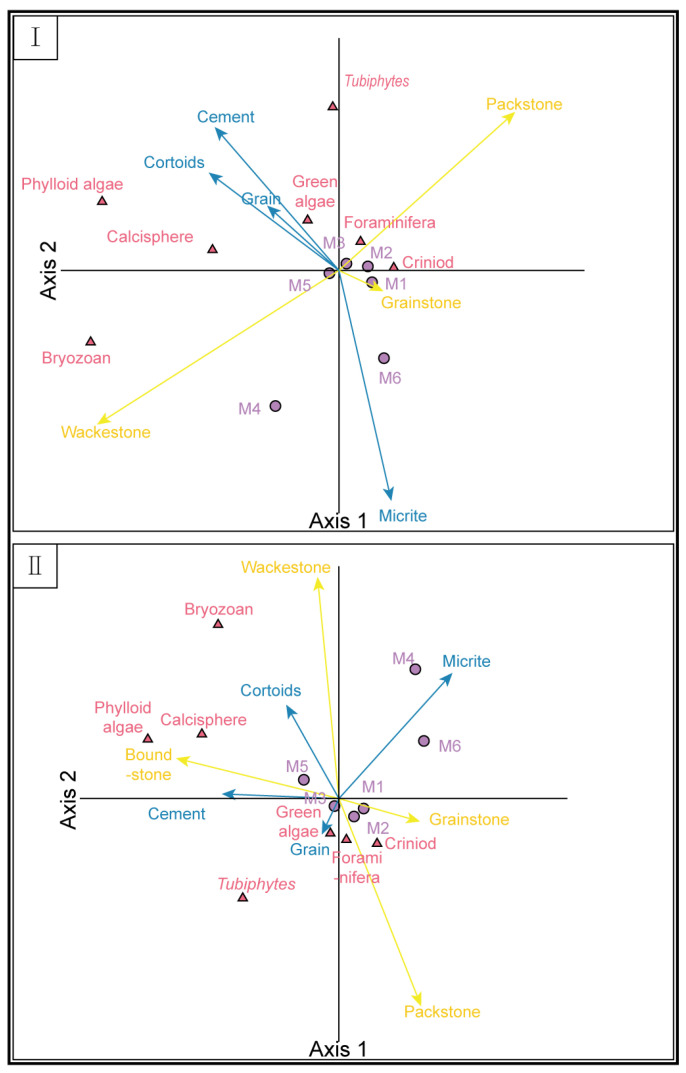
CCA triplot. (**I**) CCA triplot of non-bioconstruction. (**II**) CCA triplot of the whole section.

In environments without bioconstructions, bryozoans, phylloid algae, and *Tubiphytes* are significantly associated with environmental factors (Figure 10). They display positive correlations with an abundance of cements, grains, and cortoids. Phylloid algae and bryozoans are more strongly correlated with wackestone, whereas *Tubiphytes* are more inclined toward packstone. Within the foraminifera morphogroups, the M3 and M5 groups did not show correlations with other factors. In contrast, the M1 and M2 groups displayed weak correlations with grainstone and the contents of foraminifera and crinoids. Conversely, the M4 and M6 groups showed negative correlations with the presence of cements, grains, and cortoids but positive correlations with micrite content. The M4 group was also correlated with wackestone.

The overall environmental conditions of the entire section were similar to those of the environments excluding bioconstructions. Bryozoans, phylloid algae, and *Tubiphytes* displayed the most pronounced associations with environmental factors. Specifically, bryozoans and *Tubiphytes* were linked to wackestone and packstone, respectively, and both exhibited positive correlations with boundstone. While the M4 and M6 groups continued to exhibit positive correlations with micrite, they appeared to lack a correlation with boundstone, showing an increase in their correlation with cortoids. Additionally, the M5 group was positively correlated with boundstone.

### 4.4. Theoretical Ecospace

Since the codes for tiering and motility are ordered—with tiering codes ranging from 1 to 6 representing a gradual transition from high to low, and motility codes from 1 to 6 indicating decreasing motility—the proportion of each code can be used to calculate the average tiering and average motility for each biotic assemblage (Figure 8).

During the Moscovian to early Kasimovian, the average tiering and motility data remained relatively stable. Changes in average tiering occurred in microbial mounds, phylloid algae biostrome, and microbial biostrome, reflecting a transition from microbial-dominated communities to phylloid algae-dominated communities. The average tiering increased from 2.8 to 3.7 during this transition. However, in the early Kasimovian, significant fluctuations were observed in the phylloid algae biostrome and microbial biostrome. During the first development of the phylloid algae biostrome, the tiering data rapidly decreased from 3.9 to 3.0. As the phylloid algae biostrome transitioned back to microbial biostrome, there was a slight increase from 3.0 to 3.2. This upward trend continued as the microbial biostrome increased, ultimately increasing to 4.0 when the microbial presence ceased. Subsequently, in the phylloid algae biostrome, the average tiering quickly dropped to 3.1. From the middle to late Kasimovian, changes in the average tiering data were primarily observed in the phylloid algae biostromes, showing slight increases within a range of 3.0 to 3.5. From the late Kasimovian to the Gzhelian, the overall range of change was large, from 2.1 to 4.3, with frequent fluctuations during this period.

## 5. Discussion

### 5.1. Environment Factors

**Figure 11:**
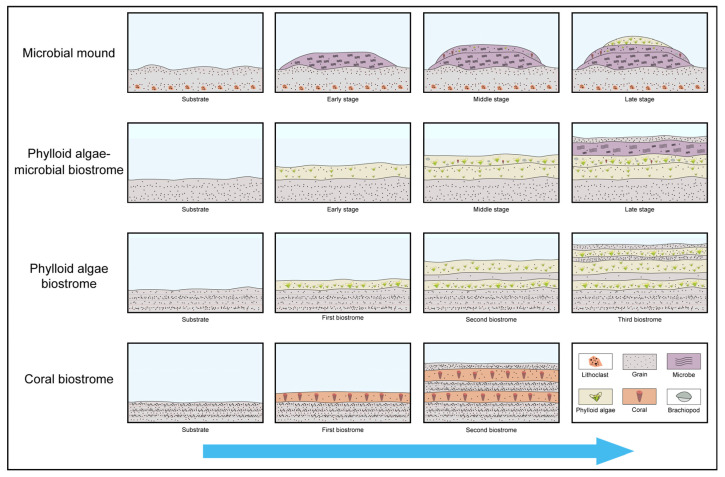
The formation and development model of bioconstructions.

On the basis of the microfacies analysis and quantitative statistics, the development and decline of bioconstructions (Figure 11) are often accompanied by environmental changes.

The lower to middle parts of the microbial mound substrate predominantly consist of lithoclastic rudstone, which formed in relatively high-energy platform margin to slope environments. In the upper part of the substrate, bioclastic packstones indicate relatively weak hydrodynamic conditions, characterized by abundant micrite and small grains, suggesting deposition below the wave base. At the top of the substrate, the increase in well-rounded grains suggests enhanced hydrodynamic conditions. In the early stage of the microbial mound, it is dominated by extensive cemented microbial boundstones, often encrusting bioclasts and lithoclasts. In the middle stage, the water continued to shallow. On the basis of the microfacies analysis, the microbial mound occurred in a shallow photic zone near the wave base, with moderate hydrodynamic conditions but not in a persistently turbulent environment. The proportion of microbial boundstones decreases. Biodiversity notably increases. Organisms such as phylloid algae, corals, bryozoans, and sponges begin to appear, without forming effective frameworks. Additionally, organisms such as crinoids, brachiopods, gastropods, and foraminifera are common and well-preserved. In the late stage, the water depth increased, represented by phylloid algae boundstone. Bioclastic packstone at the top of the microbial mound indicates a relatively turbulent aquatic environment above the wave base, marking another episode of shallowing.

The depositional environment of microbial mounds and the microbial-phylloid biostrome is similar to that of platform margin environments. The substrate of the phylloid biostromes consists of bioclastic packstone to grainstone, reflecting frequent changes in the environment. During the early stages of phylloid algae biostrome development, the thalli were relatively fragmented. In the middle stage, corals, brachiopods, gastropods, and sponges began to appear. The algae thalli became well-preserved. In the late stage, the water gradually deepened and the phylloid algae boundstone transitioned to microbial boundstone. Microbes became dominant, with a declining trend in biodiversity and abundance excluding crinoids and *Tubiphytes*. Above the phylloid algae biostrome is bioclastic packstone with abundant bioclastic fragments, suggesting a shoal environment. These findings indicate that the shallowing of relative sea level and increased hydrodynamic conditions terminated the development of the phylloid algae biostrome.

The bioconstructions developed in the mid Kasimovian are composed of three phylloid algae biostromes, separated by thin packstone-wackestone intervals. The first phylloid algae biostrome developed on a wackestone substrate. The substrate is characterized by low biodiversity with few green algae and phylloid algae, indicating an environment of relatively deep water. Compared with the substrate, the phylloid algae biostrome is located in a shallow environment. The first biostrome consists of dense and elongated algae thalli that are well-preserved. Foraminifera and *Tubiphytes* begin to appear, and their abundance increases. In the latest stage, the biostrome transitions to bioclastic wackestone, indicating a relative rise in sea level. The second phylloid algae biostrome is characterized by relatively sparse and small size algae fragments, resulting in a noticeable increase in biodiversity. Bryozoans and crinoids begin to emerge, and the abundance of foraminifera and green algae also significantly increases, with only a slight decrease in the abundance of *Tubiphytes*. The late stage of the second biostrome consists of bioclastic packstone, characterized by fragmented bioclasts, indicating a shoal environment above the normal wave base. During the early stages of the second biostrome, the relative shallowing of the water led to the termination of its growth. In the third biostrome, the microfacies indicate a platform margin environment with relatively increasing water depth. In the early stage, long and well-preserved algae thalli are frequently observed. The biodiversity remains relatively high, and most of the bioclasts in the second biostrome are also present. However, in the middle stage of the biostrome, bryozoans and *Tubiphytes* are rarely found, and the abundances of other organisms such as crinoids and foraminifera decrease significantly. During the latest stage, the water depth decreased and transitioned to a shoal environment, marking the end of the development of the third biostrome.

The coral biostromes predominantly developed within bioclastic wackestone, where fine skeletal grains and abundant micrite indicate deposition in a relatively deep, low-energy environment [40]. Bioclastic grainstone and packstone in the substrate and overlying bed are common shoal deposits that form in shallow water environments with high water energy conditions [27]. Corals thrive in relatively stable sedimentary environments despite frequent fluctuations in sea level. 

The development of bioconstructions during the Pennsylvanian was closely linked to relative sea level fluctuations and sedimentary environmental changes. Microfacies analysis revealed a dynamic interplay between high-energy and lower energy environments, reflected in the diverse lithological and biological compositions observed in microbial and phylloid algae bioconstructions. The alternating phases of shallowing and deepening water levels not only shaped the overall sedimentary environment, but also influenced the distribution and preservation of various organisms.

The CCA diagram revealed a positive correlation between the composition of cements, cortoids, and grains. This combination of factors suggests shallow marine conditions with relatively high rates of agitation [27,30]. The proportion of micrite, which is negatively correlated with the presence of cements, cortoids, and grains, is believed to indicate the accumulation of mud in a low energy or organic-rich environment [27,39]. Hydrodynamics, as a critical environmental factor in the Lumazhai section, clearly controls the distribution of organisms.

Phylloid algae, *Tubiphytes*, and bryozoans demonstrate a clear dependence on the presence of cements, cortoids, and grains, indicating their likely deposition in relatively high-energy paleoenvironments. They possess external morphology, growth habits, attachment mechanisms, and physiological characteristics that enable them to grow and reproduce in environments with relatively high hydrodynamic conditions. The increased water flow facilitates better nutrient availability, allowing these organisms to acquire essential nutrients more effectively.

In contrast, the M4 and M6 groups are positively correlated with micrite, suggesting their preference for relatively calm water environments. Although the M4 and M6 groups have been reported in both shallow-water and deep-water environments, their fragile associated tests make them less suitable for high-energy environments. As a result, their occurrence is significantly positively correlated with micrite, an indicator of low-energy environments, in the CCA. 

Despite their ability to adapt to high-energy environments, phylloid algae and bryozoans demonstrate superior growth under relatively stable low to moderate hydrodynamic conditions when correlated with wackestone. Lower hydrodynamic conditions reduce attachment difficulty, allowing phylloid algae and bryozoans to achieve more stable growth and reproduction. Consequently, they are better equipped to establish and sustain their populations and ecosystems. *Tubiphytes* have been recognized for their inherent ability to bind and immobilize other skeletal grains [69,70,71]. The presence of abundant biogenic skeletal particles within packstone provides ideal conditions for *Tubiphytes*, which explains the observed positive correlation between *Tubiphytes* and packstone. 

The CCA highlights the significant influence of hydrodynamic conditions on the distribution and growth of various organisms in the Lumazhai section. These findings underscore the critical role of hydrodynamics in shaping the ecological distribution and sedimentary characteristics within this region.

### 5.2. Average Tiering and Average Motility

The data from the average tiering (Figure 8) indicate that microbial-phylloid algae mounds and phylloid algae biostromes range from 3.1 to 3.4. The average motility data reflects values of 2.1 for microbial-phylloid algae mounds and 3.6 for phylloid algae biostrome in the early Kasimovian and 4.1–4.2 for phylloid algae biostromes in the middle Kasimovian. Despite differences in factors such as water depth and hydrodynamic conditions, the data indicate similar average tiering for organisms within the Lumazhai phylloid algae bioconstructions across varying sedimentary environments. The presence of phylloid algae is often accompanied by increased biodiversity and an abundance of skeletal grains including green algae, foraminifera, bryozoans, crinoids, and *Tubiphytes*. This trend becomes more pronounced as the phylloid algae proliferate further. However, dense growth and framework formation of the algae result in a noticeable decrease in the abundance of various bioclasts, particularly foraminifera and crinoids. The changes in phylloid algae abundance are consistent with the relatively high abundance, even during the peak development of the phylloid algae. Phylloid algae are a type of calcareous algae that can rapidly settle and expand on the seafloor [34]. Consequently, in environments conducive to phylloid algae growth, phylloid algae undergo significant development and provide favorable conditions for other organisms such as attachment substrates and protective spaces. The dense algae frameworks spatially provide analogous living conditions for other organisms, fostering the aggregation of communities.

Microfacies analyses indicate relatively low energy conditions in the microbial-phylloid algae mounds, potentially contributing to the observed variability in average motility data. Thus, phylloid algae mounds allowed for the survival and aggregation of organisms with weak mobility. In contrast, the average motility of phylloid algae biostromes under high hydrodynamic conditions is high. The changes in the abundances of *Tubiphytes* and the M5 group of foraminifera also support this point. *Tubiphytes*, with their ability to attach, maintained their high abundance in the late stage. In the CCA, the M5 group also showed a positive correlation with boundstone, likely due to their attachment behavior [67]. Moreover, the frequent relative sea-level fluctuations associated with phylloid algae biostromes may have conferred competitive advantages to organisms with strong mobility. CCA highlights the significant role of hydrodynamic conditions in the distribution of organisms in the Lumazhai section.

In the microbial mound and microbial biostrome of Lumazhai, different types of grains respond differently to the development of microbes. Concurrently, average tiering data indicate a rising trend as microbial development progressed, whereas average motility data indicate a declining trend. 

Microbes are believed to have proliferated significantly following extinction events, possibly due to the availability of ecological space resulting from the absence of metazoans [72,73,74,75]. This absence may have reduced predation, disturbance, and damage by other metazoans to the microbial mats. Throughout the development of microbial mounds, notable responses were observed in bryozoans, foraminifers, and crinoids. In the initial stages, metazoans were present at low abundance. Skeletal grains and lithoclasts provide a substrate for microbial growth, with dense microbial micrite accumulating parallel to the surface and reinforcing the framework. As microbes thrive, the previously established framework offers a suitable habitat for organisms such as foraminifers, phylloid algae, bryozoans, and crinoids. The CCA diagram revealed a significant correlation between boundstone, cortoids, and cement. The presence of abundant marine cement in the microbial mounds indicates an open sea water circulation [45], providing ample nutrient supply for suspension feeders. This results in the prolific development and increased abundance of bryozoans, crinoids, and some foraminifera. In the middle to late stages of microbial development, organisms such as corals, brachiopods, and gastropods started to appear and flourished on the periphery of the microbial mounds. With the increasing abundance of various organisms, microbes gradually decreased in abundance and eventually disappeared. In the microbial biostromes, a similar pattern can be observed. The increasing trend in average tiering data indicates a gradual expansion of ecological niches and the development of communities from the bottom to the top. The decreasing trend in average motility during microbial development also suggests that more metazoans with the ability to move tended to appear first. As microbial communities further evolved and the environment stabilized, organisms with a weak ability to move began to grow. This observation is more evident in the distribution of foraminifera abundance. In the early stages of development, mobile foraminiferal groups often dominated the total foraminifera count while other groups with relatively weaker mobility tended to appear in the middle to late stages of development.

The coral biostrome is located above the packstone and foraminiferal grainstone in the Gzhelian, with a thin thickness of less than 0.5 m. The biological community within the coral biostromes is relatively simple, primarily composed of *Fomichevella*, *Ivanovia*, foraminifers, and a small number of molluscs. There are no attached organisms such as microbes and algae. Crinoids and algae debris are typically present as granular deposits.

Figure 8 shows that both before and after the development of the coral biostromes, significant fluctuations in average tiering and average motility occurred. This finding indicates that during this period, not only did the depositional environments in the Lumazhai section change frequently, but they also lacked sustained stability throughout the ecosystem. Stable environments and community structures are critical factors in coral reef development. Hence, the frequent changes in sedimentary environments and community structures hindered the establishment of complex reef systems, leading to the formation of only coral biostromes.

## 6. Conclusions

Biological richness varies with sea level fluctuations. Additionally, the average tiering and motility of ecological communities are crucial indicators. However, the distribution of organisms, especially the factors influencing bioconstruction development, does not directly correlate with sea level changes. Observations from the Lumazhai section show that phylloid algae constructions often exhibited similar biotic community compositions in different environments, maintaining high consistency in average tiering. Microfacies analysis, ecological behaviors of high-density organisms within bioconstructions, CCA correlations, and trends in average motility indicate that hydrodynamic conditions are the primary factor influencing the development of phylloid algae constructions. During the late Kasimovian to Gzhelian, the prevalence of shoal environments and indications of high-frequency sea level changes and energetic aquatic conditions possibly contributed to the absence of phylloid algae constructions in the upper parts of the Lumazhai section. These findings suggest that despite sea level fluctuations, phylloid algae, known for rapid colonization and development, can quickly recover and regenerate, particularly during periods of frequent sea level changes.

Microbial constructions in Lumazhai flourished in diverse depositional environments. While community compositions and biological abundances varied during microbial growth, they all exhibited low biodiversity and abundance in the initial stages of development. They subsequently transitioned from more to less ecological tiers and from more to less motility during their progression. This finding is consistent with the coupling of microbial carbonate abundance fluctuations and diversity changes in metazoans throughout geological history.

The study of the Lumazhai section provides a comprehensive understanding of how sea level changes and hydrodynamic conditions interacted with biological responses, shaping the evolution of bioconstructions during the Pennsylvanian.

## Data Availability

The raw data supporting the conclusions of this article will be made available by the authors on request.
